# Health measurement using the ICF: Test-retest reliability study of ICF codes and qualifiers in geriatric care

**DOI:** 10.1186/1477-7525-3-46

**Published:** 2005-07-29

**Authors:** Jiro Okochi, Sakiko Utsunomiya, Tai Takahashi

**Affiliations:** 1 Department of Health Services Coordination, Graduate School of Medical Sciences. Kyushu University. Maedashi 3-1-1 Higashiku, Fukuoka, 812-8586, Japan; 2 Department of Health Service Management, International University of Health and Welfare, 2600-1 Kita-Kanamaru Ohtawara, Tochigi, 324-0011, Japan

## Abstract

**Background:**

The International Classification of Functioning, Disability and Health (ICF) was published by the World Health Organization (WHO) to standardize descriptions of health and disability. Little is known about the reliability and clinical relevance of measurements using the ICF and its qualifiers. This study examines the test-retest reliability of ICF codes, and the rate of immeasurability in long-term care settings of the elderly to evaluate the clinical applicability of the ICF and its qualifiers, and the ICF checklist.

**Methods:**

Reliability of 85 body function (BF) items and 152 activity and participation (AP) items of the ICF was studied using a test-retest procedure with a sample of 742 elderly persons from 59 institutional and at home care service centers. Test-retest reliability was estimated using the weighted kappa statistic. The clinical relevance of the ICF was estimated by calculating immeasurability rate. The effect of the measurement settings and evaluators' experience was analyzed by stratification of these variables. The properties of each item were evaluated using both the kappa statistic and immeasurability rate to assess the clinical applicability of WHO's ICF checklist in the elderly care setting.

**Results:**

The median of the weighted kappa statistics of 85 BF and 152 AP items were 0.46 and 0.55 respectively. The reproducibility statistics improved when the measurements were performed by experienced evaluators. Some chapters such as genitourinary and reproductive functions in the BF domain and major life area in the AP domain contained more items with lower test-retest reliability measures and rated as immeasurable than in the other chapters. Some items in the ICF checklist were rated as unreliable and immeasurable.

**Conclusion:**

The reliability of the ICF codes when measured with the current ICF qualifiers is relatively low. The result in increase in reliability according to evaluators' experience suggests proper education will have positive effects to raise the reliability. The ICF checklist contains some items that are difficult to be applied in the geriatric care settings. The improvements should be achieved by selecting the most relevant items for each measurement and by developing appropriate qualifiers for each code according to the interest of the users.

## Background

The International Classification of Functioning, Disability and Health (ICF) was published by the World Health Organization (WHO) in 2001 to standardize descriptions of health and disability[1]. Not only has the ICF provided a conceptual framework for description of functioning and disability, health professionals can use it as a tool to describe necessary information concerning people with disabilities[2]. Whence the standardization of the language is achieved with the ICF, areas of potential application include description of disability cases[3]; standardization of clinical recording systems, and comparison of disability statistics between countries[4]. As WHO has provided the ICF with its qualifier, existing health measures can be mapped to the ICF [5,6]. It may also be possible to develop measurement scales from the ICF codes[7]. The ICF consists of four domains: *body structures*, *body functions *(BF), *activities and participation *(AP), and *environment*. The term "disability" is further defined as "impairment" (dysfunction or loss of "body functions or structure"), "limitation" (the difficulty an individual may experience in executing a particular activity) or "restriction" (problems an individual may experience in involvement in life situations). Every domain of the ICF has hierarchical structure, with increasing code values (higher digit items) corresponding to more specific functions or activities. These are the characteristics of the ICF as taxonomy. In addition, WHO applied the ICF to describe the level of disability. For this purpose, WHO developed the qualifiers relevant to each domain, and they were added to the ICF codes. For example, using the *Performance *qualifier, mild restriction of "d4500 walking short distance" is coded d4500.1. According to WHO, the ICF code without qualifier does not have an inherent meaning when used for individuals or cases[8]; thus the qualifier is indispensable to denote the level of health.

The ICF in its current version consists of 1424 codes. Therefore, it is necessary to select a subset of the codes as needed for any given purpose. One of such activities is the development of the ICF checklist, which is composed of major three digit ICF items, as a practical tool to elicit and record information about an individual's functioning and disability[9,10]. Other such studies involve the development of the ICF core-sets[11]. They are developed to standardize what to measure for each chronic condition.

In addition to these studies, which are aimed at determining what to measure, it is necessary to consider how to describe health and its related status using the ICF codes. One possible approach, as taken in this study, is to apply as many ICF items as possible, as measures to describe health conditions, then to discuss the reliability and applicability of the ICF codes in a health domain such as geriatric care.

In Japan, after the implementation of the long-term care insurance (LTCI) law in 2000[12], accurate assessment of the needs of elderly clients using LTCI services became necessary. In addition, the Ministry of Health, Labor and Welfare today recommends the use of the ICF in rehabilitation care planning. Therefore, it is imperative to assess the accuracy of the application of the ICF codes in geriatric care and rehabilitation settings.

This study examines test-retest reproducibility and the clinical relevance of the ICF codes related to the geriatric care setting in the context of this background. It also aims at evaluating the content validity of the ICF checklist. The research targets the BF and AP domains only, since they contain more easily measurable items and also it was reasonable to lessen the burden of the evaluators.

## Methods

788 elderly patients (age> = 65 years) using LTCI services were selected from 5 hospitals, 29 long-term care institutions, 11 day-care centers, and 14 visiting nursing service centers. Candidates were selected without regard to age, gender, or level of function. However the participants were selected on the basis of functional stability during the one week test-retest period, and ability to give informed consent to study participation. Two independent evaluators judged the stability of candidates. Therefore, a randomization approach was not used to select study participants. Written consent was obtained from all study participants except when it was obtained from a family member by proxy in cases where the subject was unable to provide written consent by him/herself. Subjects who exhibited an acute decline in function during the course of the study were excluded from the final analysis.

Between May and October 2003, each subject was independently evaluated for numerous ICF codes by two health care professionals. The two evaluations were performed within a week of each other. In addition, all evaluators concurrently administered the Typology of the Aged with Illustrations (TAI) questionnaire, a simple illustrative assessment tool developed for care-management of the long term care insurance to assess the reproducibility of ICF items[13]. The TAI is a four-scale instrument whose reliability and validity have previously been established [14,15]. Evaluators also documented subjects' chronic medical conditions, health behaviours, and living status. Each evaluator also reported his or her own professional background and years of work experience on the same questionnaire. All evaluators were provided with a comprehensive guides to the ICF codes and qualifiers and to the TAI in advance of the assessment.

Since the ICF checklist is composed of three-digit code items (31 items in BF and 48 items in AP domain), every three digit item in the BF and AP domain was initially selected. For more detailed analysis, additional four-digit items were included, by the consensus recommendations of a panel of physiotherapists, occupational therapists, speech therapists, nurses, social workers and care-managers consulted by the authors. 85 BF items (79 three-digit and 6 four-digit) and 152 AP items (81 three-digit and 71 four-digit) ultimately composed the study instrument.

Eight BF domain chapters included were: 1. "Mental Functions" (21 codes); 2. "Sensory Functions and Pain" (15 codes); 3. "Voice and Speech Functions"(4 codes); 4. "Cardiovascular, Haematological, Immunological and Respiratory Systems" (10 codes); 5. "Function of the Digestive, Metabolic and Endocrine Systems" (10 codes); 6. "Genitourinary and Reproductive Functions" (7 codes); 7. "Neuromusculoskeltal and Movement-Related Functions" (12 codes); 8. "Functions of the Skin and Related Structures" (6 codes).

Nine AP domain chapters included were: 1. "Learning and Applying Knowledge" (15 codes); 2. "General Tasks and Demands" (4 codes); 3. "Communication" (11 codes); 4. "Mobility" (64 codes); 5. "Self-care" (20 codes); 6. "Domestic Life" (14 codes); 7. "Interpersonal Interactions and Relationships" (7 codes); 8. "Major Life Areas" (12 codes) and 9. "Community, Social and Civic Life" (5 codes). Some of the three-digit ICF codes not applicable to Japanese geriatric population, such as "riding animals for transportation" (d460) were nevertheless intentionally included in order to test whether they were correctly identified as irrelevant items.

In response to concerns raised by evaluators, illustrations to each item on the study questionnaire were added to promote efficient comprehension of each ICF item[16]. These illustrations are available on the authors' website [17].

The *body functions *qualifier is used in BF measurement in this study. Two kinds of qualifiers were used in AP measurement (the performance qualifier and the capacity qualifier). The performance qualifier describes what an individual actually performs in his or her current environment, while the capacity qualifier describes an individual's ability to execute a task or an action. In this study, the performance qualifier was used to evaluate the AP limitation or restriction. According to the WHO definition, the qualifiers were graded as follows: Level 0 indicates "no problem" (0–4% limitation or restriction); Level 1 "mild problem" (5–24% limitation or restriction); Level 2 "moderate problem" (25–49% limitation or restriction); Level 3 "severe problem" (50–95% limitation or restriction) and Level 4 "complete problem" (96–100% limitation or restriction). Levels 8 were used to describe conditions "not specific" meaning the available information does not suffice to quantify the severity of the problem.

Level 9 were used to describe conditions that were "not applicable" For example, the category *d760 *(Family relationships) is not applicable to a patient with no living family members.

**Figure 1:**
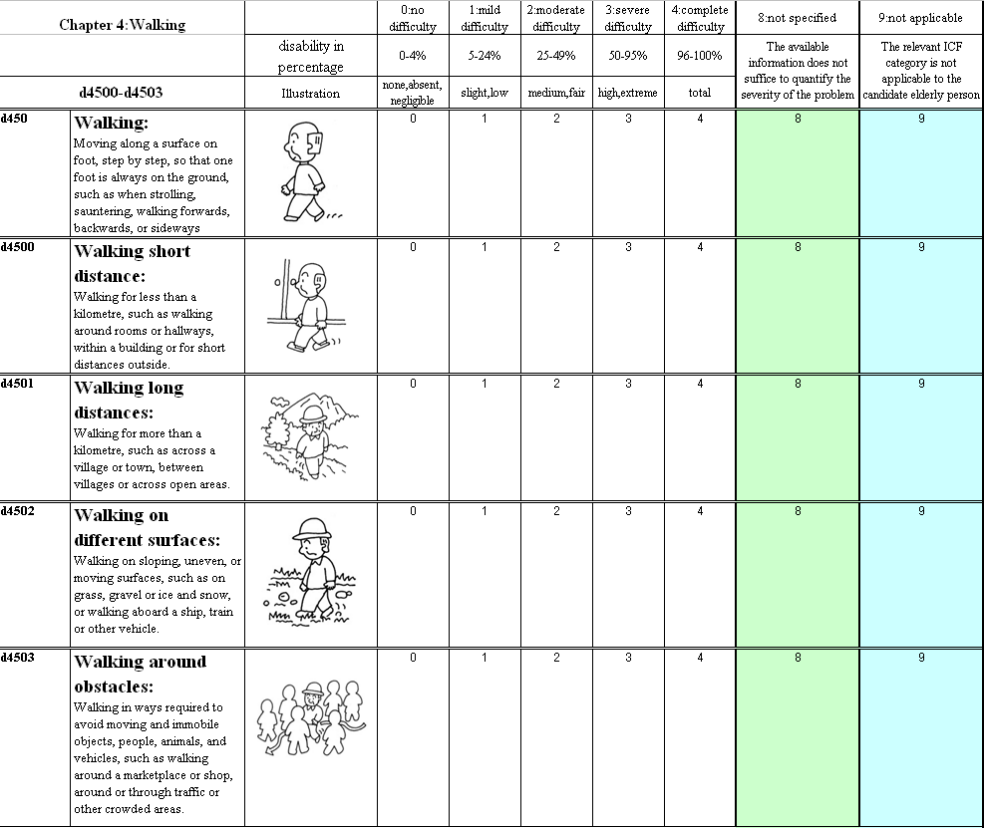
Questionnaire used in this study.

Figure 1 shows the format of the questionnaire used in this study. All the description used in the questionnaire was identical to the WHO publication on the ICF.

The evaluators were given instructions by the authors, using a manual comparable to the ICF checklist [9]. The evaluators are asked to evaluate using all possible information available, including interviews, proxy, and medical records.

To evaluate the reliability of each ICF item, the weighted kappa of each item was calculated using the data obtained by the two independent evaluators [18]. To estimate the reliability between the two, the required number of pairs is 86 with alpha and beta error level of 0.05 and 0.20 respectively, with a minimum required kappa level of 0.4 and acceptable kappa level of 0.6 [19]. Stratification was done so that there were more than 86 relevant data both in terms of measurement settings and evaluators' experience. In this study, the weighted kappa was classified according to Landis *et al *to moderate (0.41–0.60), substantial (0.61 to 0.8), and almost perfect agreement (above 0.8) [20].

The kappa value was further evaluated by stratifying the experience of the evaluators and care settings. In evaluation by box plot, the kappa statistics that showed negative values were replaced to 0.

As an index of irrelevance of the ICF codes, the immeasurability rate of each item was calculated using the sum of samples judged "non specific" or "not applicable" as the numerator and the total number of evaluations as the denominator. The properties of each item were evaluated by using the values of the weighted kappa and the immeasurability rate of each item. The analyses were performed with STATA (version 8.17).

## Results

Evaluations were performed on a total of 788 participants. Among these evaluations, one of two evaluations was not complete in 46 persons (6%). These cases were excluded from the analysis. Thus, two sets of data were independently obtained from 742 geriatric subjects yielding a total of 1484 data sets. 25% of subjects were male (mean age 78.8 years; SD = 9.2 years) and 75% were female (mean age 84.1; SD = 7.6 years); 593 were institutionalized (evaluated at the residential institution) and 149 lived at home (75 evaluated at day-care services and 74 evaluated at home).

289 experienced care professionals served as evaluators: nurses (24%), therapists (26%), care managers (22%) and social workers/caregivers (28%). The average amount of work experience as health professional was 10 years (SD8) with a median of 8 years. Among the measurements, 227 pairs were performed by evaluators who both had 8 years or more experience. Conversely, 205 pair of measurements were performed by evaluators who both had less than 8 years of experience.

### Test-retest reliability of the ICF items

The result distribution and rating as non specific (n.s), not applicable (n.a), and weighted kappa of 85 BF items (79 three-digit items and 6 four-digit items) and 152 AP items (81 three-digit items and 71 four-digit items) are shown in Additional files 1 (BF domain) and 2 (AP domain).

Weighted kappa values of BF domain items ranged from 0.13 to 0.72, with an average of 0.46 and a median of 0.44, while that of AP domain items ranged from -0.17 to 0.79, with an average of 0.55 and a median of 0.59.

Table 1 shows the weighted kappa for the BF and AP domains stratified by evaluators' years of experience, and by care settings of the samples, along with the referential weighted kappa value of the TAI scales. The institutionalized care setting showed higher average and median kappa values, compared to the setting of living at home. Average and median of the evaluation performed by a more experienced pair of evaluators exceeded those performed by a less experienced pair. The kappa values of the four TAI scales concurrently measured with the ICF items were as follows: "Mobility" 0.80 (95% C.I. 0.75–0.84); "Mental function" 0.75 (0.70–0.80); "Toileting" 0.76 (0.71–0.82); "Eating" 0.78 (0.73–0.83). The weighted kappa value of the TAI scales did not show marked differences between care settings and evaluators' experience.

**Table 1:** Average and median weighted kappa values, by care settings and evaluator experience

			care setting		evaluators experience	
		Total	institutional	At home	<8 years	> = 8 years
BF domain	(84 items)					
	Average	0.46	0.47	0.37	0.41	0.58
	SD	0.12	0.12	0.14	0.15	0.10
	Median	0.44	0.44	0.35	0.41	0.59
AP domain	(137 items)					
	Average	0.58	0.58	0.51	0.54	0.63
	SD	0.09	0.10	0.11	0.13	0.11
	Median	0.59	0.59	0.51	0.56	0.64
TAI*						
	Mobility	0.80	0.80	0.76	0.78	0.83
	Mental function	0.75	0.75	0.70	0.81	0.77
	Eating	0.76	0.76	0.77	0.82	0.79
	Toileting	0.78	0.77	0.79	0.79	0.82

*Typology of the aged with illustrations

The higher average kappa value of measurement in institution is not likely due to the experience of the evaluator, since the measurement performed at home contained more pairs of evaluation by experienced evaluators (69%).

**Figure 2:**
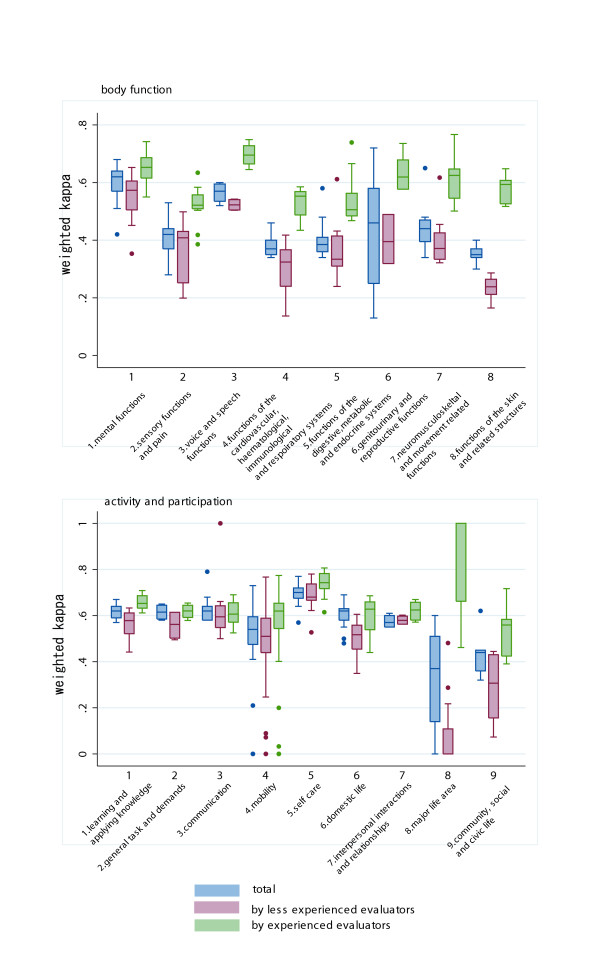
Box plot of weighted kappa of the ICF BF domain by chapter.

**Figure 3:**
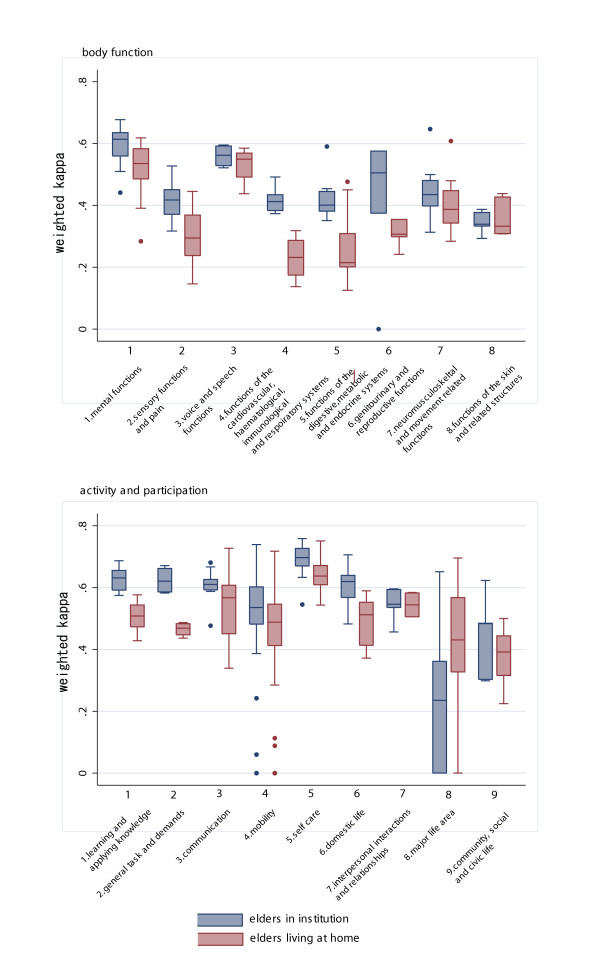
Box plot of weighted kappa of the ICF AP domain by chapter.

Figure 2 shows the box plot of weighted kappa statistics by chapters of the BF and AP domains respectively. Chapter 2,4,5,6 and 7 in the BF domain and Chapters 8 and 9 in the AP domain showed relatively low reliability. In the BF domain the weighted kappa result by the pair of experienced evaluators showed better measurement reproducibility for all chapters. In AP the domain, experienced evaluators showed better reproducibility in chapters 1,5,6,7,8 and 9,

Figure 3 shows the box plot of weighted kappa stratified by care setting. Most of the chapters, except for the chapter 8 and 9 of the AP domain, showed higher reliability. However, caution must be paid when interpreting the weighted kappa result in chapter 8, because kappa values were far less accurate when the results were stratified.

### Immeasurability rate

The immeasurability rate of BF domain items ranged from 0.00 to 0.96, with an average of 0.06 and a median of 0.03. That of the AP domain items ranged from 0.00 to 0.90, with an average of 0.13 and a median of 0.02.

**Figure 4:**
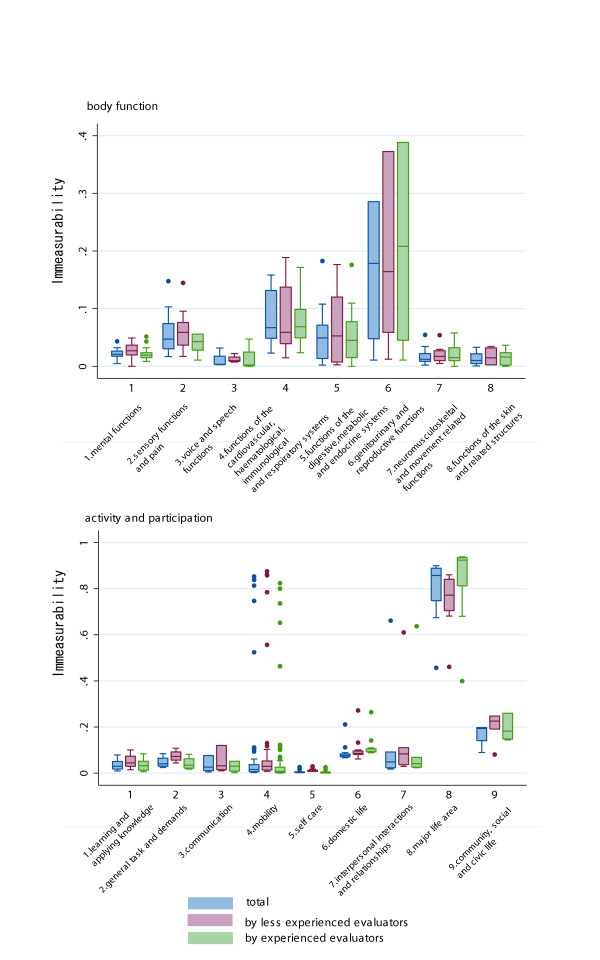
Box plot of immeasurability rate of the ICF BF domain by chapter.

Figure 4 shows the box plot of the immeasurability stratified by the evaluators' years of experience. In the BF domain, the highest immeasurability item was "sexual functions" (b640): 0.96. Because this is an exceptionally large figure, it was not plotted on Figure 4. Other top five items rated as immeasurable within the BF domain were "menstruation functions" (b650): 0.28, "sensations associated with genital and reproductive functions" (b670): 0.26, "endocrine gland functions" (b555): 0.18, "procreation functions" (b660): 0.18. Except for the item "endocrine gland functions" (b555), all fell within chapter 6 of BF domain, "Genitourinary and Reproductive Functions". The top 5 items rated as immeasurable in AP domain were "preschool education" (d815): 0.90; "school education" (d820): 0.90; "higher education" (d830): 0.89; "producing messages in formal sign language" (d340): 0.89; and "vocational training" (d825): 0.89. Except for the item "communicating in formal sign language" (d340), all fell within chapter 8 of the ICF AP domain, "Major Life Areas". The pattern of immeasurability by chapter did not differ according to the evaluators' experience.

**Figure 5:**
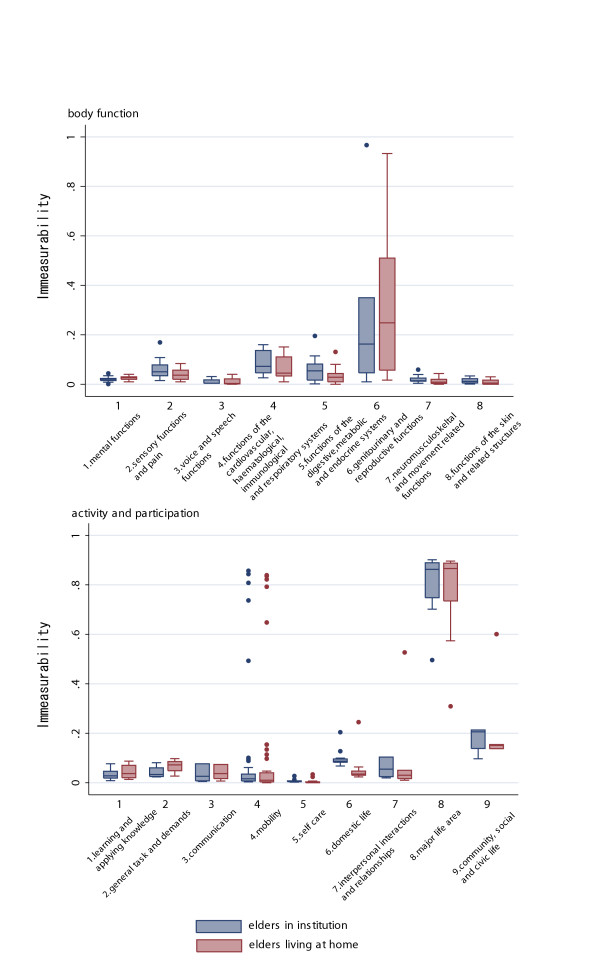
Box plot of immeasurability rate of the ICF AP domain by chapter.

Figure 5 shows the immeasurability rate by care settings of elderly persons. Except for the immeasurability rate of BF domain chapter 6 (domestic life) which showed a lower immeasurability rate compared to the elderly in the institutional settings, there were no marked differences in immeasurability rate by care setting.

### Properties of the ICF items

The weighted kappa statistics and the immeasurability rate of three-digit AP and BF domain codes are categorized as shown in Additional file 3 and 4 respectively. Items were classified into 3 categories: high reliability (weighted kappa ≥ 0.6); intermediate reliability (0.4 ≤ weighted kappa<0.6); and low reliability (weighted kappa < 0.4) using all data. Items were secondly categorized by the median value of the immeasurability rate (immeasurability ≥ 0.3 for the BF domain: immeasurability ≥ 0.2 for the AP domain, the median score) or of low requirement (immeasurability <0.3 for the AP domain: immeasurability rate < 0.2 for the AP domain). Additionally, each item was flagged as to whether it was included in the ICF checklist.

High reliability and measurable items included in the study instrument, but not found in the ICF Checklist were: "global psychosocial functions" (b122); "temperament and personality function"(b126); "calculation functions"(b172); "mental function of sequencing complex movements" (b176); "articulation functions"(b320) and "gait pattern functions"(b770) in BF domain, and "focusing attention" (d160); "making decisions" (d177); "transferring oneself" (d420); "Moving around in different location" (d460) in the AP domain. On the contrary, items evaluated as low reliability and immeasurable in the ICF Checklist were: "blood pressure functions"(b420); "Haematological system functions"(b430); "immunological system functions"(b435); "respiration functions"(b440)"; digestive functions" (b515); endocrine gland functions(b555) and "sexual functions" (b640) in BF domain, and "school education" (d820); "apprenticeship"(d840); "religion and spirituality" (d930) and "human rights"(d940) in the AP domain.

## Discussion

The clinical application of ICF codes to diverse populations remains an active topic of discussion [21-27], with little consensus as to how each code and qualifier must be utilized for specific populations. There are related previous studies, which deal with the concept of ICF model using different exisiting scales [28-30]. Some studies dealt with the ICF reproducibility to assign ICF categories to extant measures[3]. In geriatric care research, Jette *et al*. have identified distinct concepts shared by activity and participation[31].

However, still to date, to the best of the authors' knowledge, there is no study that has shown the test-retest reproducibility of the ICF as a scale to evaluate functioning in a specific population.

The ICF is based on a universal model that theoretically can be applied regardless of cultures, age groups or care settings [7,27,32]. However, various codes may have different implications for various care settings in practical terms, and individual ICF items requires validity and reliability studies in application to diverse populations. Such efforts are already underway in the form of development of ICF core-sets for specific medical conditions[11]. Conceptual applications of the ICF to National surveys have also been undertaken[4,33,34].

This study differs from both these approaches, as it does not rely on the experts' opinions to assure face and content validity, but applies the ICF directly as an instrument of geriatric assessment to select more adequate items, while aiming to develop new scales using ICF taxonomy.

It requires a certain level of test-retest reproducibility and measurability, or discard of items which are not appropriate to create new scales.

The authors are now developing the elderly communication performance scale according to the result of test-retest reliability statistics, because AP items related to communications have acceptable level of test-retest reliability.

Items such as d320 and d340, which are related to communication using formal sign language, showed low measurability. These items are not always applicable in the general geriatric care setting, but are pertinent for individuals with hearing loss. Thus, the scale developer can select ICF items with certain reliability and measurability according to the scope of each scale.

The other rationale of testing such a wide range of the ICF codes is that elderly persons hold problems that cover multiple disciplines. This contrasts with the ICF core-set project which is relatively disease focused.

### Reliability of the ICF qualifiers

Our findings raise concerns about the low reliability of the ICF items using qualifiers.

Although overall reliability of the ICF items was low, it had improved considerably, when the weighted kappa statistics were stratified by the work experience of the evaluators. As shown in Table 1, the weighted kappa of the TAI scales did not show marked differences compared to the ICF items. A previous study on the TAI scales also indicated that the reliability was not dependent on the experience of the evaluators [15]. It indicates the ICF items and its qualifiers may be too difficult to quantify in some cases.

By stratifying the results by care-settings, it was possible to get better test-retest reproducibility in the institutional setting. This may be because more information, including medical records, are available in the setting.

The result of reliability differs depending on the chapter. As shown in Figures 2 and 3, the low weighted kappa value of chapters 4, 5 and 8 of the BF domain, and chapters 8 and 9 of the AP domain contribute to the overall low reliability of the ICF.

In BF domain, chapter 4("Functions of the Cardiovascular, Haematological, Immunological and Respiratory Systems"), 5 ("Functions of the Digestive, Metabolic and Endocrine Systems") and 8 (Functions of the Skin and Related Structures") are composed of items that can be described with specific medical examination.

For example, "blood pressure functions" (b420) can be described much more easily with blood pressure level measurable with arm cuff than using qualifier levels from 0 to 4.

### Immeasurability of the ICF items

What we call immeasurable in this study include level 8 – not specified (available information does not suffice to quantify the severity of the problem) and level 9 – not applicable (e.g., *d760, Family relationships *is not applicable to an elderly person without family).

For example, in case of the global psychosocial functions (b122:immeasurability rate 2.6%), 38 evaluators could not quantify it because the sufficient information was not available and one evaluator rated it as not applicable as shown in additional file 1. This indicates that items with low immeasurability rate can be easily evaluated.

In contrast, 96% of the measurement was rated as immeasurable in sexual function (b640), and most of them were rated as not applicable, as expected by the target sample of this study. Overall, most of the rating as immeasurable was by level 8 (not specific), although some items such as chapter 8 ("Major life area") of the AP domain showed more level 9 than level 8.

Chapter 8 ("Major life area") is comprised of the categories "education" (d810-d839), "work and employment" (d840-d859), and "economic life" (d860-d879), while Chapter 9 (Community, social and civic life) includes "community life" (d910), "recreation and leisure" (d920), "religion and spirituality" (d930), "human rights" (d940) and "political life and citizenship" (d950). To accurately assign scores in the sub-domains of education, work and employment, community life, and political life in a population of institutionalized elderly patients may be difficult, or even inappropriate. Thus, the large proportion of institutionalized geriatric patients in our study sample may have affected the high immeasurability scores in these two chapters. The measurement of "religion and spirituality" and "human rights" requires multidimensional and subjective assessment. Thus it is difficult to assign either of them into a single code[35,36].

The low reliability shown in this study indicates the difficulty of using the ICF as a measurement tool and is also attributable to the ambiguous nature of the qualifiers. For example, when an evaluator judges the performance level of school education, he or she may assess the subject as level 4 ("complete difficulty"), because of the subject's inability to obtain further education or to attend an institution for learning. However, this item may also be regarded as "not applicable" or "not specified," especially in the context of institutionalized geriatric patient for whom school attendance is not an expected component of daily life.

In contrast, frequently assessed items in the LTCI assessment appeared to have high reliability. Presumably because items such as toileting and self-dressing constitute a part of a standard self-care assessment already widely used by healthcare professionals [37]. This similarity may explain the high reproducibility of self-care item assessments between independent evaluators in our study.

### Validity of the ICF Checklist

An additional purpose of this study was to evaluate the validity of the ICF Checklist in geriatric assessment. We have also used the checklist as a training tool for evaluators, because it was the sole available material at the commencement of this study for official training of the ICF. We have found that the existing ICF Checklist lacks several items which we found scored high in reliability and low in immeasurability rate. These items include, "global psychosocial functions" (b122); "temperament and personality function" (b126); "calculation functions" (b172); "mental function of sequencing complex movements" (b176); "articulation functions" (b320) and "gait pattern functions" (b770) in the BF domain, and "focusing attention" (d160); "making decisions" (d177); "transferring oneself" (d420); "Moving around in different locations" (d460) in the AP domain.

The ICF checklist includes less reliable and immeasurable items, e.g. "blood pressure functions" (b420); "haematological system functions" (b430); "immunological system functions" (b435); "respiration functions" (b440)"; digestive functions" (b515); "endocrine gland functions" (b555) and "sexual functions" (b640) in the BF domain, and "school education" (d820); "apprenticeship" (d840); "religion and spirituality" (d930) and "human rights" (d940) in the AP domain. Some of the body function related items could be better described with chronic disease, such as high blood pressure, anemia, and diabetes. Items not relevant to the elderly care settings such as school education; apprenticeship might be just omitted when applying the scheme to those settings.

Importance of participation in religions and spirituality might vary depending on cultural settings. Also, human rights (d940) may play a pivotal role on understanding geriatric domestic violence.

This result should help selecting more useful sets of the ICF items that would reflect evaluators' needs and reliability of items. Some modification to the ICF checklist may also facilitate the use of the ICF.

### Study Limitations

There are a few limitations in this study. The samples were selected from various service providers based on the stability of the function during the test-retest period. The kappa statistic is dependent on the samples. Therefore these samples might not fully represent the target population, namely the elderly using long-term care services in Japan. However, the use of a large sample obtained from multiple centers is nevertheless indicative of relatively low reliability of the ICF items measured with the qualifiers.

Also, other possible confounders such as the cultural settings and evaluators' professional backgrounds may influence the ICF measurement values. It is possible that some of the ICF items show different item functioning (DIF) depending on these confounders. The Rasch measurement technique is applicable to answer this question, which remains to be studied[38]. The illustrations added by the authors to clarify the definition of each item could have biased the results. However, our intention in incorporating illustrations was to standardize evaluator assessments. Previous studies have shown that illustrations increase the reliability of assessment instruments[39].

Lastly, the authors used the sum of qualifiers 8 and 9 as a simple index of immeasurability. Items with a high prevalence of level 8 suggested that it was difficult for the evaluator to ask the question or obtain the information from the medical chart. In contrast, assignment of a qualifier of 9, which was more prevalent in chapter 8 of AP domain, suggested these items were not applicable. However, these two qualifiers may convey quite different information, and the study design made it difficult to compare the differences between these two qualifiers. In addition, it was difficult to analyze inter-rater reliability of qualifiers 8 and 9 because of the skewed distribution of the result between these qualifier levels. However, the prevalence of these qualifiers, as shown in Additional files, should help in selecting ICF items for future research.

## Conclusion

The reliability of the ICF codes as measured with qualifiers is relatively low, and the ICF Checklist requires modification. Improvements should be achieved by selecting the most relevant items for each measurement and constructing appropriate qualifiers for each code according to the interest of users.

## Authors' contributions

JO, SU and TT carried out the study design, data collection, statistical analysis and preparation of this manuscript.

## Supplementary Material

Additional File 1 Weighted kappa statistics and immeasurability rate of the ICF BF items Click here for file

Additional File 2 Weighted kappa statistics and immeasurability rate of the ICF AP items Click here for file

Additional File 3 Property of the three-digit ICF BF items according to weighted kappa, immeasurability, and the ICF checklist Click here for file

Additional File 4 Property of the three-digit ICF AP items according to weighted kappa, immeasurability, and the ICF checklist Click here for file
